# Re-Emergence of HMPV in Gwangju, South Korea, after the COVID-19 Pandemic

**DOI:** 10.3390/pathogens12101218

**Published:** 2023-10-04

**Authors:** Sun-Ju Cho, Sun-Hee Kim, Hongsu Lee, Yeong-Un Lee, Jeongeun Mun, Sujung Park, Jungwook Park, Ji-Su Park, Kwangho Lee, Cheong-mi Lee, Jinjong Seo, Yonghwan Kim, Yoon-Seok Chung

**Affiliations:** 1 Division of Emerging Infectious Disease, Department of Infectious Disease Research, Health and Environment Research Institute of Gwangju, Gwangju 61954, Republic of Korea; sj0426@korea.kr (S.-J.C.); lhs9213@korea.kr (H.L.); gloryw3@korea.kr (Y.-U.L.); mjo3214@korea.kr (J.M.); sujung9421@korea.kr (S.P.); jwpvet@korea.kr (J.P.); nyoil658@korea.kr (J.-S.P.); twilight0930@korea.kr (K.L.); thefirstlee@korea.kr (C.-m.L.); sjj21@korea.kr (J.S.); vetkyh@korea.kr (Y.K.); 2 Division of High-Risk Pathogen, Bureau of Infectious Diseases Diagnosis Control, Korea Disease Control and Prevention Agency (KDCA), Cheongju 28159, Republic of Korea

**Keywords:** human metapneumovirus, non-pharmaceutical interventions, whole-genome sequencing

## Abstract

The non-pharmaceutical interventions implemented to prevent the spread of COVID-19 have affected the epidemiology of other respiratory viruses. In South Korea, Human metapneumovirus (HMPV) typically occurs from winter to the following spring; however, it was not detected for two years during the COVID-19 pandemic and re-emerged in the fall of 2022, which is a non-epidemic season. To examine the molecular genetic characteristics of HMPV before and after the COVID-19 pandemic, we analyzed 427 HMPV-positive samples collected in the Gwangju area from 2018 to 2022. Among these, 24 samples were subjected to whole-genome sequencing. Compared to the period before the COVID-19 pandemic, the incidence rate of HMPV in 2022 increased by 2.5-fold. Especially in the age group of 6–10 years, the incidence rate increased by more than 4.5-fold. In the phylogenetic analysis results, before the COVID-19 pandemic, the A2.2.2 lineage was predominant, while in 2022, the A2.2.1 and B2 lineage were observed. The non-pharmaceutical interventions implemented after COVID-19, such as social distancing, have reduced opportunities for exposure to HMPV, subsequently leading to decreased acquisition of immunity. As a result, HMPV occurred during non-epidemic seasons, influencing the age distribution of its occurrences.

## 1. Introduction

Human metapneumovirus (HMPV) causes respiratory infections in infants and young children (<5 years old) [1,2]. These infections are like those caused by the human respiratory syncytial virus (HRSV), ranging from upper respiratory distress to bronchiolitis and pneumonia among infants, young children, older adults, and immunocompromised hosts [3,4,5]. HMPV infection disrupts dendritic cell activity and reduces antigen-specific T cell activation, resulting in incomplete virus clearance and an increased likelihood of re-infection [6]. Thus, individuals infected with HMPV do not acquire lifelong immunity to the virus, and re-infection occurs [7,8,9].

HMPV is a non-segmented single-stranded RNA virus belonging to the family *Pneumoviridae*. The HMPV genome is approximately 13 kb long and consists of eight genes that encode nine proteins (N, P, M, F, M2-1, M2-2, SH, G, and L) [10,11]. N, P, L, M2-1, and M2-2 are proteins associated with the assembly of the nucleocapsid. The M protein envelops the RNA core, and the exterior of the virus consists of a lipid envelope. The envelope contains three surface glycoproteins: fusion protein (F), small hydrophobic protein (SH), and attachment glycol protein (G) [6].

Based on the antigenicity of the surface proteins, HMPV has been classified into two lineages: A and B. The two lineages have each been subdivided into sub-lineages, with A1, A2, B1, and B2 [10]. The A2 sub-lineage has been further subdivided into A2a and A2b [12]. Subsequently, an A2 genotype with 180-nucleotide duplication and 111-nucleotide duplication in the G protein was reported in Japan and has also been reported in Spain, China, and Croatia [13,14,15,16,17]. The strain containing a duplication in the G protein is gradually increasing [18,19]. However, the sub-classification of the A2 genotype has shown inconsistency in nomenclature among researchers [20,21,22,23]. Establishing a consistent classification system for the A2 lineage is essential for understanding the emergence of new lineages and conducting epidemiological and surveillance studies of HMPV [11].

Recently, there has been a study proposing the classification of HMPV based on genetic clustering of the F gene sequence [24]. According to this research, HMPV can be categorized into six genetic lineages: A1, A2.1, A2.2.1, A2.2.2, B1, and B2. HMPV with the presence of G protein 180-nucleotide duplication and 111-nucleotide duplication belongs to the A2.2.2 lineage. HMPV genotypes co-circulate during the epidemic season; however, no specific dominant genotypes have been identified [25,26,27], and the association between the HMPV genotype and disease severity is unclear [28].

Non-pharmaceutical interventions were implemented in response to the global COVID-19 pandemic, and these interventions may affect the circulation of other seasonal respiratory viruses [29,30]. Indeed, in South Korea as well, there have been delayed outbreaks of PIV3 after the COVID-19 pandemic. We published the research results on this last year [31]. HMPV also exhibits a seasonal distribution. It predominately occurs in the winter to spring months in the northern hemisphere and southern hemisphere [6]. The off-season outbreaks of HMPV have been reported in other countries as well following the COVID-19 pandemic. Delayed HMPV outbreaks occurred in Israel from May to June 2021; in Australia, a summer surge followed by a delayed winter season outbreak was observed from the end of 2020; in 2021, off-season outbreaks of HMPV were reported in children and adults in the UK in June and July, and in Spain, an HMPV outbreak in children was reported in November 2021 [9,32,33,34]. Similarly, In South Korea, HMPV was prevalent from late winter to spring prior to the COVID-19 pandemic but was not detected during the COVID-19 pandemic in 2020 or 2021, and an off-season HMPV outbreak was observed in the fall of 2022.

Understanding the relationship between the off-season outbreak of HMPV and the virus’ characteristics is necessary to predict and develop preventive measures against future HMPV epidemics. Molecular epidemiology studies of HMPV have focused on the G protein and the F protein [35]. Partial genome sequencing cannot capture changes occurring outside the targeted regions, which could include important viral variations. Especially, there is a possibility of missing variations like the recently reported G protein’s 180-nt duplication or 111-nt duplication using partial genome sequencing. However, whole genome sequencing allows for a comprehensive understanding of the molecular epidemiology of the entire virus [24]. Therefore, we conducted a phylogenetic analysis of the whole genome of HMPV to analyze the molecular epidemiological characteristics of HMPV before and after the COVID-19 pandemic.

## 2. Materials and Methods

### 2.1. Surveillance and Sample Collection

The Korea Influenza and Respiratory Virus Surveillance System (KINRESS) is a program overseen by the Korea Disease Control and Prevention Agency (KDCA), in which primary hospitals nationwide are selected for sampling surveillance. Every week, samples from acute respiratory patients are collected and sent to the designated research institutions for analysis. We participated in KINRESS to monitor Acute Respiratory Infections (ARIs) in South Korea. Throat or nasopharyngeal swabs were collected from outpatients with ARIs throughout the year from three primary collaborating hospitals in the Gwangju area. From 2018 to 2022, a total of 6,334 samples were collected, and we conducted screening for eight types of acute respiratory viruses, including HMPV.

### 2.2. RNA Extraction and Real-Time PCR

Following the manufacturer’s instructions, nucleic acids were extracted from the samples using a QIAamp RNA kit (Qiagen, Hilden, Germany). We used 140 µL samples and 60 µL final nucleic acid elutions.

HMPV was identified by using a One-step RSV A&B/HMPV Real-time PCR Kit (Kogene, Seoul, Korea) in accordance with the manufacturer’s instructions. The amplification conditions were as follows: 50 °C for 30 min, followed by 95 °C for 10 min and 40 cycles of 95 °C for 15 s and 60 °C for 1 min.

### 2.3. Spcemens and Samples Collection

A total of 427 HMPV-positive samples were collected from the Gwangju area during 2018–2022. To investigate the age distribution of HMPV occurrences, we divided the samples into a total of seven groups: 0–2 years, 3–5 years, 6–10 years, 11–20 years, 21–40 years, 41–60 years, and >60 years.

### 2.4. Whole Genome Sequencing

Among the 427 HMPV-positive samples, a total of 24 samples for whole-genome sequencing were randomly selected based on Ct values of ≤25. Out of the 24 samples, 16 were from the COVID-19 pandemic, and eight were after the COVID-19 pandemic samples. Viral RNA was extracted using a QIAamp Viral RNA mini Kit (Qiagen, Hilden, Germany) according to the manufacturer’s instructions. A panel was developed using Ion AmpliSeq On-Demand Panel (ThermoFisher Scientific, Carlsbad, CA, USA) technology for use on the Ion Torrent platform (ThermoFisher Scientific, Carlsbad, CA, USA). The customized panel was designed to obtain coverage of the entire HMPV genome using combinations of 100 and 125 primers divided into two sets (pools) according to the manufacturer’s protocol. Reverse transcription was performed using a SuperScript VILO cDNA Synthesis Kit (Thermo Fisher Scientific, Carlsbad, CA, USA) following the manufacturer’s recommendations. For library preparation, an Ion Ampliseq Library 2.0 Kit (Thermo Fisher Scientific, Carlsbad, CA, USA) was used according to the manufacturer’s protocol. The automated Ion Chef (ThermoFisher Scientific, Carlsbad, CA, USA) instrument prepared templates from the 25 μL sample pool using Ion 510/520/530 Chef Kits and Ion 530 Chips that were sequenced using the Ion S5 XL sequencer (ThermoFisher Scientific, Carlsbad, CA, USA). The sequencing reads were aligned, mapped, and subjected to variant analysis using the CLC Genomics Workbench 21.0.3 (Qiagen, Hilden, Germany) program.

### 2.5. Phylogenetic Analyses

For the phylogenetic analyses, 53 reference strains were selected from GenBank (Table S1). Multiple sequence alignment was performed using the MUSCLE algorithm in MEGA X software. Phylogenetic trees were constructed using the Maximum Likelihood (ML) method with the General Time Reversible model in MEGA X software. The reliability of the branching order was assessed by performing 1000 bootstrap replicates.

## 3. Results

### 3.1. Epidemiology of HMPV

**Figure 1:**
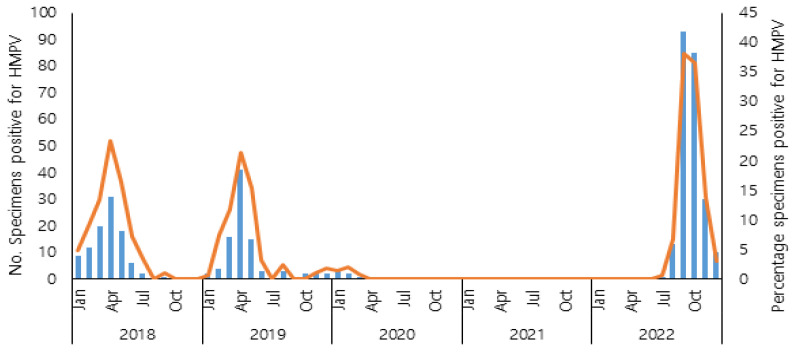
Seasonality pattern of Human metapneumovirus (HMPV) positive cases from 2018 to 2022 in Gwangju, South Korea.

Before the COVID-19 pandemic, human metapneumovirus exhibited a progressive increase from January, reaching its peak in April, followed by a subsequent decline during the summer months. During the COVID-19 pandemic, HMPV infections rarely occurred in 2020, and HMPV was not detected in 2021, coinciding with the implementation of non-pharmacological interventions against COVID-19. In 2022, according to the results of the KINRESS in the Gwangju area, HMPV reappeared in July, and the number of HMPV-positive cases increased in September and October. The incidence rate of HMPV before and after the COVID-19 pandemic showed that the incidence rate of HMPV after COVID-19 was 2.5 times higher than before COVID-19 and increased significantly. The seasonal distribution of HMPV infections from 2018 to 2022 is shown in Figure 1. The age distribution of HMPV occurrences showed an approximately 2-fold increase in the age groups 0–2 years and 3–5 years. Particularly, there was a more than 4.5-fold increase in the age group 6–10 years, and this increase was statistically significant (Table 1). However, there was no significant difference in the incidence rate of HMPV between males and females before and after the COVID-19 pandemic (Table 1).

### 3.2. Phylogenetic Analysis of HMPV Whole Genome Sequences

**Figure 2:**
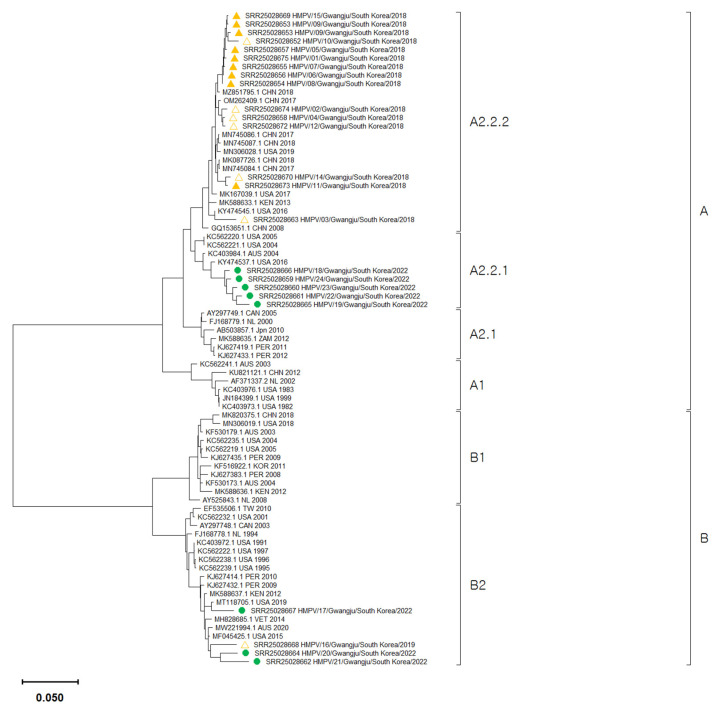
The phylogenetic tree was constructed based on 77 whole HMPV genome sequences. The tree was created using the maximum likelihood method with a GTR+G+I substitution model and tested with 1000 bootstrap replicates. In the tree, HMPV samples before the pandemic were depicted as triangles. Among them, samples containing a 111-nucleotide duplication in the G gene were indicated with orange triangles. HMPV samples after the pandemic were represented by green circles. All sequences from this study have been registered in the SRA (Sequence Read Archive) database (accession number: PRJNA987724, https://www.ncbi.nlm.nih.gov/sra/PRJNA987724, it can be accessed after 31 July 2024). Biosample accession numbers of all strains are indicated in parentheses.

We analyzed 24 whole-genome sequences and 53 reference sequences obtained from GenBank to determine their lineages. Among the 16 before the COVID-19 pandemic samples, 15 clustered within the A2.2.2 lineage, and one belonged to the B2 lineage. Out of the 15 samples belonging to the A2.2.2 lineage, 9 were strains carrying a 111-nucleotide duplication in the G protein. Among the eight strains identified after the COVID-19 pandemic, five belonged to the A2.2.1 lineage, and three belonged to the B2 lineage. A1, A2.1, and B1 were not detected in any of the samples analyzed in this study. The 2022 A2.2.1 sequences were observed in a monophyletic clade, with one sequence that circulated in the USA in 2016. However, the 2022 B2 sequences were distributed between two closely related strains, one from Australia in 2020 and the other from the USA in 2019, without clade formation (Figure 2). The whole genome sequences of this study have been deposited in the NCBI Sequence Read Archive under the Bio Project PRJNA987724.

## 4. Discussion

The epidemiological patterns of human metapneumovirus (HMPV) in Korea signify its role as a noteworthy respiratory pathogen. HMPV is recognized for inducing acute respiratory infections, especially among young children and the elderly. Within Korea, HMPV infections exhibit seasonal trends, demonstrating elevated incidence rates during colder months, generally from late fall to early spring. The virus is frequently linked to respiratory symptoms, including fever, cough, and breathing difficulties.

The non-pharmaceutical interventions implemented to prevent COVID-19, such as mandatory mask-wearing, social distancing, and travel restrictions, have affected the prevalence of respiratory viruses [29,30]. Social distancing measures were implemented in South Korea following the first COVID-19 outbreak in January 2020 and relaxed by April 2022. Changes in the prevalence of respiratory viruses were also observed during this period. According to the KINRESS results, PIV3, which did not occur in 2020, re-emerged in the fall of 2021 [31]. In 2022, HMPV reappeared in the fall, which is typically a non-epidemic season, and the magnitude of the epidemic was larger than that before the COVID-19 pandemic.

HMPV mainly affects children under five years of age [1,32]. In this study, we also observed a significant increase in HMPV incidence among children aged 5 and under before and after the COVID-19 pandemic, consistent with the known pattern of HMPV primarily affecting this age group [1,32]. However, after the COVID-19 pandemic, there was a significant increase in the age group of 6–10 years affected by HMPV. An atypical age distribution of acute respiratory viruses after the COVID-19 pandemic has also been observed in RSV [36,37]. After the COVID-19 pandemic, HMPV did not occur for two years. Children aged 5 and under who had spent two years with lowered immunity to HMPV, were exposed to HMPV after the COVID-19 pandemic, leading to an increase in the age of occurrence beyond 5 years. In other words, this atypical age distribution might be associated with reduced immunity owing to the lack of exposure to HMPV during the COVID-19 pandemic.

This study conducted a whole-genome analysis to investigate the molecular genetic characteristics of HMPV before and after the COVID-19 pandemic. The whole genome sequencing of viruses using NGS as a pathogen has enabled comprehensive molecular epidemiological analysis [35,38,39]. Furthermore, using highly detailed molecular biological classification, it is highly useful for understanding the characteristics of viruses with a high level of resolution [24]. Analysis of the 24 whole-genome sequences confirmed the circulation of HMPV lineages A2.2.1, A2.2.2, and B2 between 2018 and 2022. Before the COVID-19 pandemic, strains from the A2.2.2 and B2 lineages circulated, and among the 15 strains from the A2.2.2 lineage, 9 had a 111-nt duplication in the G protein. After the COVID-19 pandemic, strains from the A2.2.1 and B2 lineages circulated. Although the number of analyzed samples was limited, and definitive conclusions cannot be drawn, it seems that the circulating lineages changed between before and after the COVID-19 pandemic.

Although some researchers have suggested that the A2.2.2 lineage may be more virulent, the variation in virulence among HMPV lineages remains unclear [14,40,41]. In this study, the A2.2.1 lineage was responsible for the re-emergence in 2022, and the scale of occurrence was larger than that before the COVID-19 pandemic when the A2.2.2 lineage was prevalent. This could be due to lower herd immunity to the HMPV virus resulting from reduced exposure during social distancing measures rather than differences in virulence between the lineages.

In this study, all genotype B strains belonged to the B2 lineage. Out of the 24 strains, lineage A strains were detected more often than lineage B viruses. This could have been due to sample bias selection. The selection of samples was based on Ct values ≤25, and previously, it was shown that this could result in a biased selection of the samples (Groen et al. [24]).

Overall, due to the social distancing measures implemented to prevent the spread of COVID-19, there was a lack of exposure to HMPV, resulting in lower natural immunity to HMPV. With the relaxation of social distancing measures in 2022, HMPV exposure during this period led to an off-season outbreak of HMPV in South Korea. This indicates that the COVID-19 pandemic may have impacted the age and lineage distribution of HMPV, emphasizing the importance of social distancing measures. Strengthening herd immunity is thought to help prevent future epidemics. Therefore, continuous genomic monitoring of HMPV is required for vaccine development and distribution.

## 5. Conclusions

Although the sample size was limited, and definitive conclusions cannot be drawn from this study, the data suggest differences in the circulation of HMPV lineages before and after the COVID-19 pandemic. In addition, a significant increase in HMPV-positive patients was identified in individuals aged 6–10 years after the COVID-19 pandemic compared with the period before the pandemic. This might be explained by the implementation of social distancing measures during the pandemic, which reduced the exposure to HMPV in younger children and established herd immunity. The release of the measurements has subsequently led to off-season outbreaks of not only HMPV infections but also other respiratory infections.

## Figures and Tables

**Table 1:** Age and sex distribution of HMPV-positive samples from 2018 to 2022 in Gwangju, South Korea.

Variable	2018–2020 (n = 195)			2022 (n = 232)			*p*-Value ^1^
	Number of Patients	Number of HMPV-Positive	Prevalence of HMPV(%)	Number of Patients	Number of HMPV-Positive	Prevalence of HMPV(%)	
	4264	195	4.6	2070	232	11.2	<0.01 *
Sex							0.389
Male	1939	86	4.4	985	112	11.4	
Female	2325	109	4.7	1085	120	11.1	
Age							
0–2 years	690	33	4.8	666	65	9.8	<0.01 *
3–5 years	954	77	8.1	481	96	20.0	<0.01 *
6–10 years	705	32	4.5	204	43	21.1	<0.01 *
11–20 years	444	13	2.9	209	10	4.8	0.164
21–40 years	463	6	1.3	194	9	4.6	0.012
41–60 years	451	13	2.9	145	6	4.1	0.305
60–90 years	557	21	3.8	171	3	1.8	0.947

^1^ Chi-square test among 2018, 2019, 2020, and 2022. * *p* < 0.01.

## Data Availability

The dataset generated for this study can be found online. All sequences from this study have been registered in the SRA (Sequence Read Archive) database (accession number: PRJNA987724).

## Supplementary Materials

The following supporting information can be downloaded at: https://www.mdpi.com/article/10.3390/pathogens12101218/s1, Table S1: Accession numbers of viruses used as reference for phylogenic analysis of the HMPV-positive samples.
