# Supplementary material for: Re-Emergence of HMPV in Gwangju, South Korea, after the COVID-19 Pandemic
Source: Pathogens. 2023 Oct 4;12(10):1218. doi: 10.3390/pathogens12101218 (PMC10609798; doi:10.3390/pathogens12101218)
Supplement: Supplementary file 1 [file pathogens-12-01218-s001.zip › pathogens-2557366-supplementary.pdf]

Table S1. Accession numbers of viruses used as reference for phylogenic analysis of the HMPV-positive samples.

Reference Virus Accession No.	Country/Year
AB503857.1	Japan/2010
AY297748.1	Canada/2003
MK820375.1	China/2018
AY297749.1	Canada/2005
EF535506.1	Taiwan/2010
GQ153651.1	China/2008
MK588633.1	Kenya/2013
MK588635.1	Zambia/2012
MK588636.1	Kenya/2012
JN184399.1	USA/1999
KC403972.1	USA/1991
KC403973.1	USA/1982
KC403976.1	USA/1983
KC403984.1	Australia/2004
KC562219.1	USA/2005
KC562220.1	USA/2005
KC562221.1	USA/2004
KC562222.1	USA/1997
KC562232.1	USA/2001
KC562235.1	USA/2004
KC562238.1	USA/1996
KC562239.1	USA/1995
KC562241.1	Australia/2003
KF516922.1	Korea/2011
KF530173.1	Australia/2004
KF530179.1	Australia/2003
KJ627383.1	Peru/2008
KJ627414.1	Peru/2010
KJ627419.1	Peru/2011
KJ627432.1	Peru/2009
KJ627433.1	Peru/2012
KJ627435.1	Peru/2009
KU821121.1	China/2012
KY474537.1	USA/2016
MK087726.1	China/2018
MK167039.1	USA/2017
MK588637.1	Kenya/2012
MN306019.1	USA/2018
MN306028.1	USA/2019
MN745086.1	China/2017
MN745087.1	China/2018

AF371337.2	Netherlands/2002
FJ168779.1	Netherlands/2000
AY525843.1	Netherlands/2008
FJ168778.1	Netherlands/1994
MZ851795.1	China/2018
OM262409.1	China/2017
MN745084.1	China/2017
KY474545.1	USA/2016
MW221994.	Australia/2020
MT118705.1	USA/2019
MH828685.1	Vietnam/2014
MF045425.1	USA/2015
